# The association between lifestyle factors, and physical and mental health in inpatients with MI: a network analysis

**DOI:** 10.1192/j.eurpsy.2022.1576

**Published:** 2022-09-01

**Authors:** N. Den Bleijker, M. Van Schothorst, I. Hendriksen, W. Cahn, J. Deenik

**Affiliations:** 1 GGz Centraal, Scientific Research Department, Amersfoort, Netherlands; 2 GGz Centraal, Wetenschappelijk Onderzoek, Amersfoort, Netherlands; 3 LivIng Active, -, Santpoort-Zuid , Netherlands; 4 University Medical Centre Utrecht, Psychiatry, Utrecht, Netherlands

**Keywords:** Lifestyle, physical health, mental health

## Abstract

**Introduction:**

People with mental illness (MI) have a reduced life expectancy compared to the general population, mostly attributable to somatic diseases caused by poor physical health. Lifestyle factors (exercise, sleep, diet, substance use) are associated with poor physical and mental health. Although lifestyle factors, and physical and mental health are believed to be interconnected, research has mainly focused on one-sided relationships. Currently, we are implementing a lifestyle focussed approach in treatment, in which we assess lifestyle factors as well as physical and mental health of people with MI on a large scale (˜850 places of residence).

**Objectives:**

To investigate the association between lifestyle factors, and physical and mental health in people with MI.

**Methods:**

Baseline data from an open cohort cluster randomized stepped wedge study. Lifestyle factors (exercise, sleep, diet, substance use), physical health, medication use and psychological health (symptoms, quality of life) were assessed using data from patient files and questionnaires. Associations will be analysed with network analyses.

**Results:**

First results (N≈1600) show that 54% of patients have high blood pressure, 51% have excessive waist circumference, 46% are experiencing sleep problems, 71% smoke and 88% do not meet exercise guidelines. Patients experience a lower quality of life compared to the general population.

**Conclusions:**

Initial results show that patients have poor physical health, low quality of life and an unhealthy lifestyle. Further analyses are currently being conducted to gain insight in the complex pattern between lifestyle factors, and physical and mental health. This can contribute to the improvement of routine clinical care.

**Disclosure:**

No significant relationships.

